# Preliminary study on the effects of boysenberry juice intake on brown adipose tissue activity in healthy adults

**DOI:** 10.1038/s41598-024-76452-4

**Published:** 2024-10-24

**Authors:** Ryo Furuuchi, Satoshi Kato, Daisuke Maejima, Tatsuro Amano, Shinya Fujiki, Ippei Shimizu, Tohru Minamino

**Affiliations:** 1 Advanced Research Institutes, Bourbon Corporation, 316-2 Higashijima, Akiha-ku, Niigata city, Niigata 945-0841 Japan; 2 Department of Advanced Senotherapeutics, Juntendo University Graduate School of Medicine, Tokyo, Japan; 3 Laboratory for Exercise and Environment Physiology, Faculty of Education, Niigata University, Niigata, Japan; 4 Department of Cardiovascular Medicine, Niigata University Graduate School of Medical and Dental Sciences, Niigata, Japan; 5 Department of Cardiovascular Aging, National Cerebral and Cardiovascular Center Research Institute, Osaka, Japan; 6 Department of Cardiovascular Biology and Medicine, Juntendo University Graduate School of Medicine, Tokyo, Japan

**Keywords:** Brown adipose tissue, Anthocyanin, Boysenberry, Thermogenesis, Fat oxidation, Nutrition, Nutritional supplements, Translational research

## Abstract

Brown adipose tissue (BAT) plays an important role in energy metabolism because it uses fatty acids for thermogenesis during cold exposure. Preclinical studies found that boysenberry anthocyanins (BoyACs) activate BAT. Therefore, the aim of this preliminary study was to evaluate how BoyAC intake affects BAT in humans. We performed an open-label single-arm nonrandomized study in healthy volunteers. Before and after 4 weeks of daily consumption of 100 ml boysenberry juice (BoyJ) containing 61 mg of BoyACs, participants were assessed at 24 °C and then after 1 h of mild cold exposure (18 °C). An infrared thermography camera was used to measure skin surface temperatures in the supraclavicular BAT region (Tscv) and the non-BAT region of the upper chest (Tch). Energy metabolism was measured by indirect calorimetry. For each endpoint, we calculated Δ as the difference between values before and after cold exposure and compared the values before and after BoyJ intake. 10 volunteers participated (age: 36.1 ± 4.1, body mass index (BMI): 20.9 ± 0.6). After BoyJ intake, ΔTscv-ch was significantly higher (*p* = 0.029), but Δ energy expenditure, Δ fat oxidation, and Δ carbohydrate oxidation were not significantly different. We found a significant positive correlation between BMI and Δfat oxidation with BoyJ intake. The results indicate that 4 weeks of BoyJ intake activates cold-induced thermogenesis in the scv-BAT but does not have a significant effect on energy metabolism. BoyJ intake may increase fat oxidation during cold exposure in individuals with higher BMI.

*Trial registry number*: UMIN000043476, 05/03/2021.

**Supplementary Information:**

The online version contains supplementary material available at 10.1038/s41598-024-76452-4.

## Introduction

The primary function of brown adipose tissue (BAT) is non-shivering thermogenesis. In cold conditions, mitochondria in BAT produce heat by oxidizing fatty acids, preventing an attenuation of body temperature^1^. This process is especially important in neonates^2^and hibernating animals^3^, but studies have recently reported that it contributes to the maintenance of body temperature even in human adults^4,5^. BAT consumes energy for thermogenesis and thus increases the energy metabolic rate, so it may potentially play an important role in weight management and obesity prevention^6^. Several studies have shown that BAT activation improves insulin sensitivity and has a positive effect on glucose metabolism^7^. Activated BAT tissue is expected to consume excess lipids and reduce low-density lipoprotein cholesterol in the blood, and those effects may help to reduce the risk of cardiovascular disease^8^. BAT activity is known to decrease with aging and obesity^9^, and activation of BAT is thought to be important for the prevention and treatment of metabolic syndrome^10^.

In recent years, interest has grown in the influence of foods on BAT activation. For example, capsinoids^11^, omega-3 fatty acids^12^, and some polyphenols^13^have been reported to enhance energy metabolism by activating BAT. Furthermore, in preclinical studies, we showed that boysenberry anthocyanins (BoyACs), the major component of the juice from boysenberries (a hybrid Rubus berry of Rubus baileyanus and Rubus loganobaccus that contains multiple polyphenols)^14^, protect BAT from metabolic stress^15^. Other studies have also reported that anthocyanins activate BAT^16,17^. Most of the studies on the relationship between food and BAT were preclinical, and an insufficient number of studies have evaluated the effects of foods on human BAT. In particular, to our knowledge no study has investigated whether anthocyanins affect BAT in humans. Therefore, we think that to evaluate the relationship between anthocyanins intake and human BAT is necessary for real-world applications. We hypothesized that BoyACs intake would activate BAT and alter energy metabolism during cold exposure and performed a preliminary human intervention study to evaluate the effects of BoyACs on human BAT.

## Results

### Baseline characteristics

**Fig. 1:**
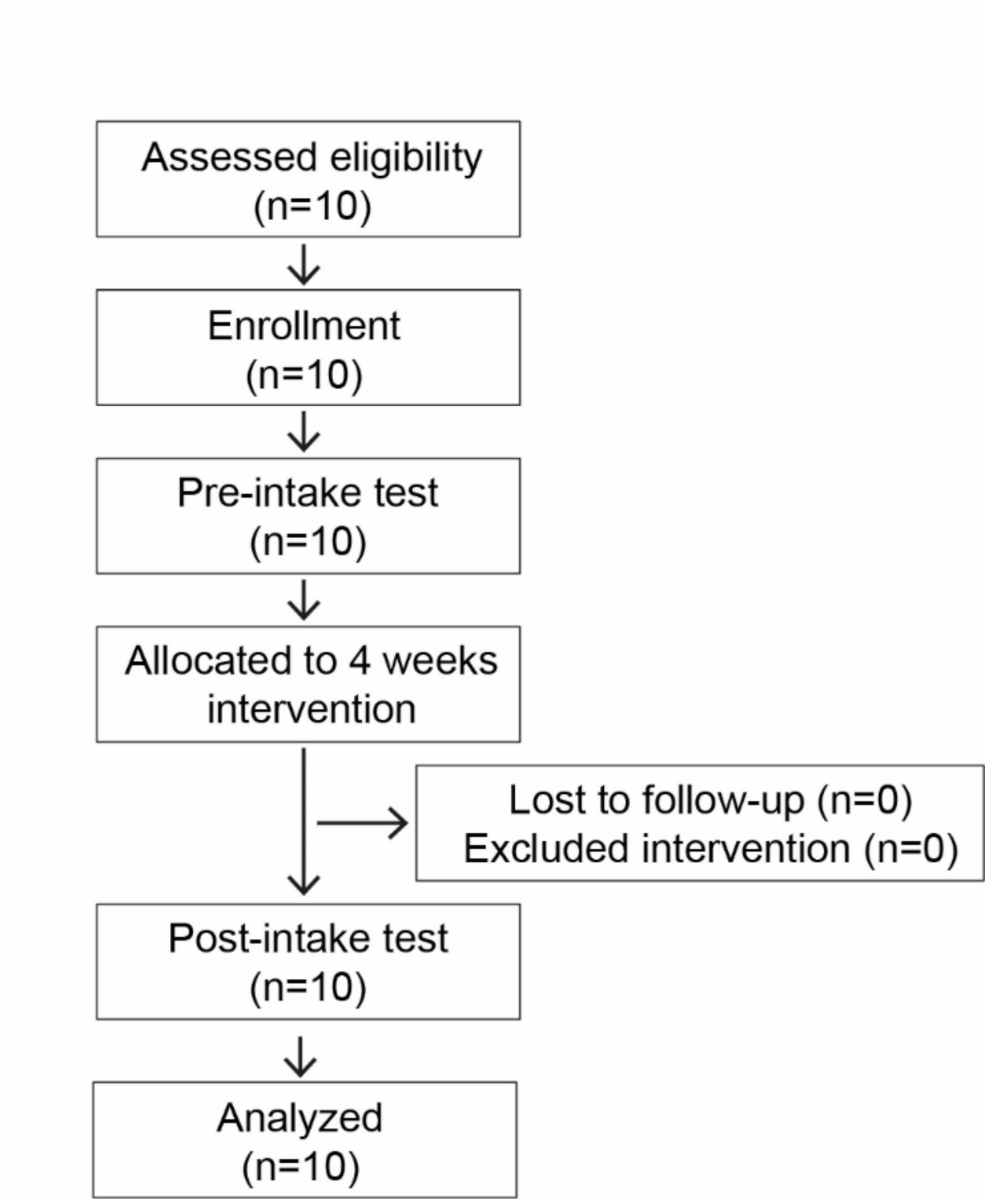
Study flow diagram. Ten subjects participated in the study. All subjects completed the study and all data were used for analysis.

Ten volunteers, aged between 20 and 57 years, agreed to participate in the study, and at screening, all 10 were eligible for the study. Participant baseline characteristics are shown in Table 1. None of the participants discontinued or dropped out of the study, and all 10 were included in the analysis. The study flow diagram is shown in Fig. 1. There were no significant changes in height, body weight, or body mass index (BMI) from before to after intake of BoyJ.

**Table 1:** Participant characteristics.

	Pre-intake	Post-intake	*p* value
Age	36.1 ± 4.1		
Male/Female	4/6		
Height (cm)	163.8 ± 2.8	163.8 ± 2.8	0.622
Body weight (kg)	56.5 ± 3.0	56.2 ± 2.9	0.332
BMI (kg/m^2^)^a^	20.9 ± 0.6	20.8 ± 0.6	0.365

Data represent the mean ± SEM. The two groups were compared by a paired *t* test. The raw data are shown in Supplementary Data S1.

^a^ Body mass index (BMI) is weight (kilograms) divided by height (meters) squared.

### Skin surface temperature

**Fig. 2:**
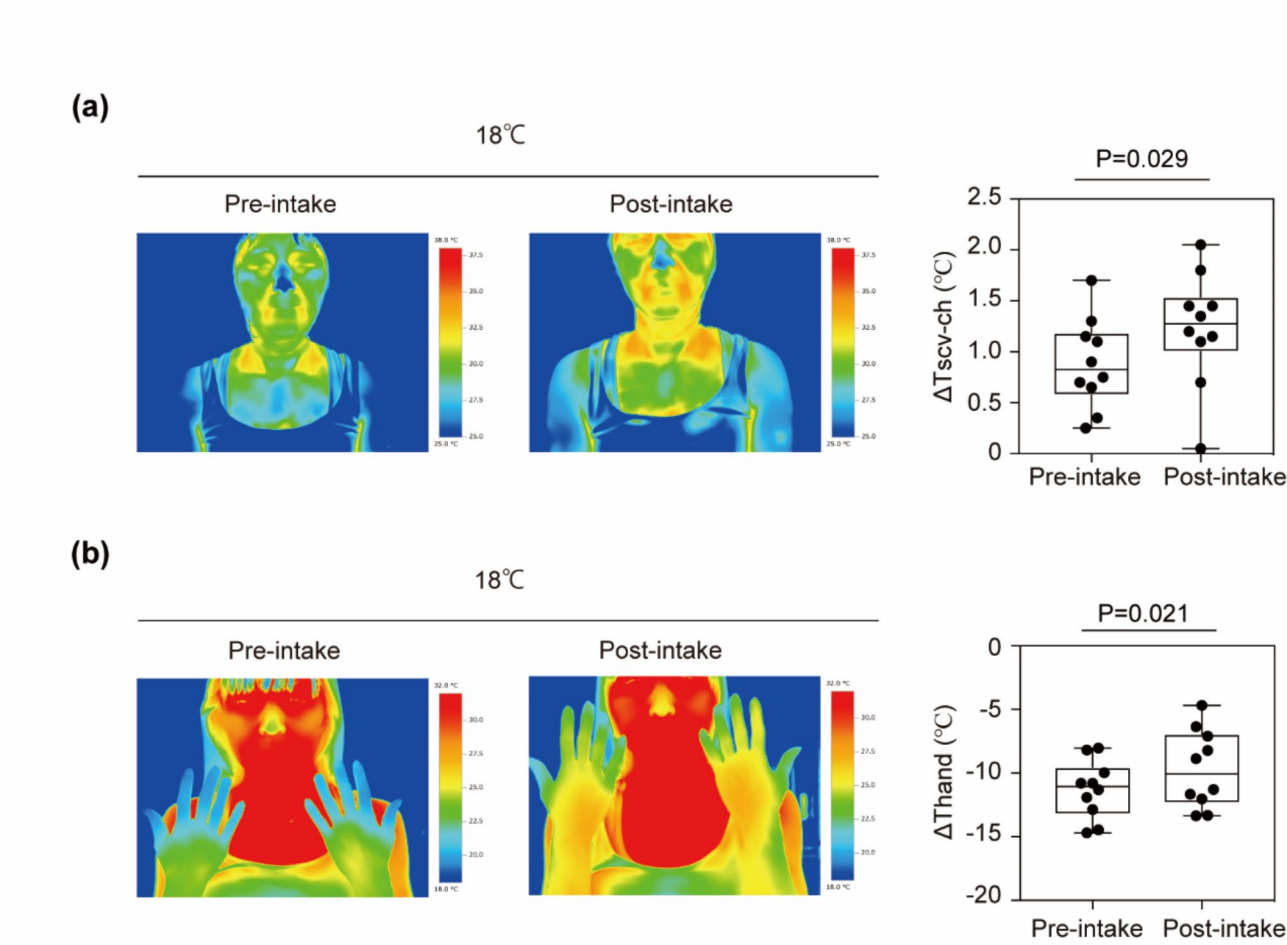
Change in skin surface temperature. (**a**) Boysenberry juice (BoyJ) intake increased the temperature of the supraclavicular brown adipose tissue (BAT) region (Tscv) and hand skin surface after cold exposure (18 °C) for 1 h. The data show the difference (Δ) in skin surface temperature when participants were exposed to 24 °C and 18 °C. Representative infrared thermography images of Tscv and the non-BAT region in the upper chest (Tch) during cold exposure. The graph shows the difference between Tscv and Tch (ΔTscv-ch). (**b**) Representative infrared thermography images of the hand skin surface. The graph shows the difference in hand temperature when participants were exposed to 24 °C and 18 °C. A paired *t* test was used to compare hand temperature before and after intake of BoyJ; p values are shown in the figure. The raw data are shown in Supplementary Data S2.

Skin surface temperature measurements are shown in Table 2. ΔTscv-ch, i.e., the activity of the BAT region, increased significantly from 0.89 °C ± 0.14 °C before BoyJ intake to 1.23 °C ± 0.18 °C after 4 weeks of intake (*p* = 0.029, Fig. 2a). On the other hand, no significant difference was found for Tscv or Tch (*p* = 0.329 and *p* = 0.958, respectively). Thand increased significantly from − 9.56 ± 0.49 °C before intake of BoyJ to -7.88 ± 0.67 °C after intake (*p* = 0.021, Fig. 2b).

**Table 2:** Change in skin surface temperature before and after 4 weeks’ intake of boysenberry juice.

		Pre-intake	Post-intake	*P* value
Tscv (℃)^a^	24 °C	34.6 ± 0.1	34.7 ± 0.3	0.329
	18 °C	32.9 ± 0.4	33.3 ± 0.4	
	Δ	-1.68 ± 0.33	-1.35 ± 0.32	
Tch (℃)^b^	24 °C	33.3 ± 0.1	33.4 ± 0.3	0.958
	18 °C	30.7 ± 0.4	30.8 ± 0.5	
	Δ	-2.56 ± 0.36	-2.58 ± 0.40	
Tscv-ch (℃)^c^	24 °C	1.29 ± 0.09	1.27 ± 0.14	0.029
	18 °C	2.17 ± 0.19	2.50 ± 0.22	
	Δ	0.89 ± 0.14	1.23 ± 0.18	
Thand (℃)^d^	24 °C	31.4 ± 0.5	31.7 ± 0.6	0.021
	18 °C	21.8 ± 0.4	23.8 ± 0.6	
	Δ	-9.56 ± 0.49	-7.88 ± 0.67	

Data represent the mean ± SEM. Δ represented by subtracting the value at 24 °C from the value at 18 °C. The two groups were compared by a paired *t* test. The raw data are shown in Supplementary Data S2.

^a^Skin surface temperature of the supraclavicular. ^b^Skin surface temperature of the upper chest. ^c^Difference between Tscv and Tch. ^d^Skin surface temperature of the hand.

### Energy metabolism

**Fig. 3:**
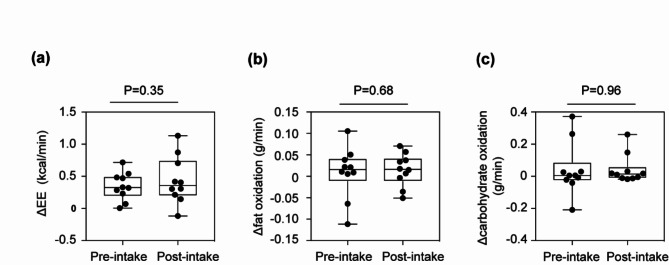
Changes in energy metabolism. Boysenberry juice (BoyJ) had no significant effect on energy metabolism. The data show the difference (Δ) in energy metabolism when participants were exposed to 24 °C and 18 °C. Change (Δ) in energy expenditure (Δ EE, **a**), Δ fat oxidation (**b**), and Δ carbohydrate oxidation (**c**) were determined by indirect calorimetry measurements. A paired *t* test was used to compare hand temperature before and after intake of BoyJ; p values are shown in the figure. The raw data are shown in Supplementary Data S3.

The results of the respiratory gas analysis are shown in Table 3, and the values of Δ EE, Δ fat oxidation, and Δ carbohydrate oxidation, which were analyzed to estimate the effects of cold exposure on BAT activation, are shown in Fig. 3. No significant changes were found in Δ EE (pre-intake, 0.35 ± 0.07 kcal/min; post-intake, 0.44 ± 0.12 kcal/min; *p* = 0.350, Fig. 3a), Δ fat oxidation (pre-intake, 0.0087 ± 0.0189 g/min; post-intake, 0.0148 ± 0.0120 g/min; *p* = 0.677, Fig. 3b), or Δ carbohydrate oxidation (pre-intake, 0.042 ± 0.051 g/min; post-intake, 0.044 ± 0.028 g/min; *p* = 0.959, Fig. 3c).

**Table 3:** Change in energy metabolism from before to after 4 weeks’ intake of boysenberry juice.

		Pre-intake	Post-intake	*P* value
EE (kcal/min)	24 °C	1.31 ± 0.11	1.21 ± 0.08	0.350
	18 °C	1.65 ± 0.16	1.65 ± 0.18	
	Δ	0.35 ± 0.07	0.44 ± 0.12	
Fat oxidaition (g/min)	24 °C	0.0419 ± 0.0087	0.0379 ± 0.0047	0.677
	18 °C	0.0506 ± 0.0190	0.0526 ± 0.0154	
	Δ	0.0087 ± 0.0189	0.0148 ± 0.0120	
Carbohydrate oxidation (g/min)	24 °C	0.137 ± 0.023	0.129 ± 0.014	0.959
	18 °C	0.179 ± 0.053	0.173 ± 0.041	
	Δ	0.042 ± 0.051	0.044 ± 0.028	

Data represent the mean ± SEM. Δ represented by subtracting the value at 24 °C from the value at 18 °C. The two groups were compared by a paired *t* test. The raw data are shown in Supplementary Data S3. EE, energy expenditure.

**Fig. 4:**
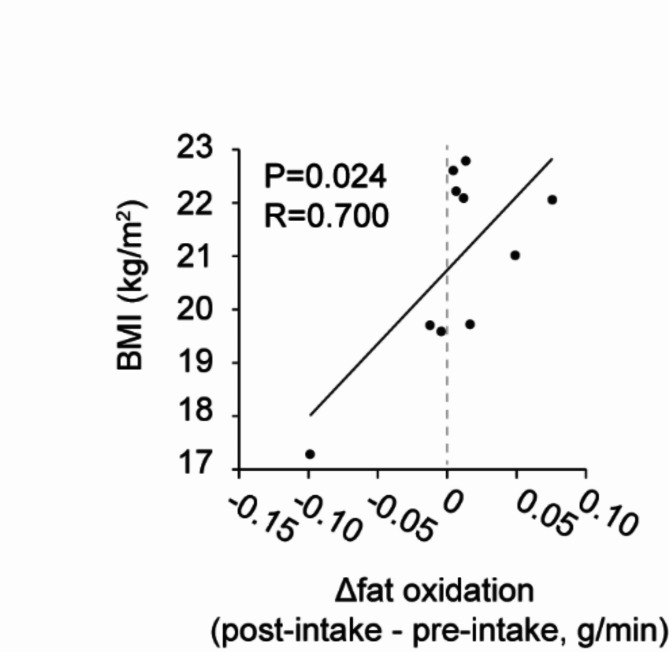
Correlation between body mass index (BMI) and fat oxidation. BMI was positively correlated with increased cold-induced fat oxidation after 4 weeks of Boysenberry juice (BoyJ) consumption. The change in fat oxidation from before to after cold exposure (18 °C) was compared before and after 4 weeks’ intake of BoyJ. A Pearson correlation test was used to calculate the r and p values.

An exploratory evaluation of the correlation between each data revealed a positive correlation between Δ fat oxidation (post-intake – pre-intake) and BMI (Fig. 4, *r* = 0.700, *p* = 0.024). No significant correlations were observed between the other data.

A post hoc power calculation for fat oxidation was performed with paired and two-sided t tests and a significance level of 5%. When all participants were included (*N* = 10; mean difference, 0.00105 [SD, 0.0370]), the power was calculated to be 0.051 and the effect size 0.028, indicating that the power was insufficient. On the other hand, when only participants with a BMI of 20 or more were included (*N* = 6; mean difference, 0.0173 [SD, 0.0392]), the power increased to 0.144 and the effect size to 0.442.

### Cold sensation

Changes in the cold sensation as assessed by the VAS are shown in Table 4. The VAS score for Δ cold sensation did not change significantly from before consumption of BoyJ (50.6 ± 10.7 mm) to after (50.5 ± 8.7 mm; *p* = 0.994).

**Table 4:** Change in cold sensation assessed by a visual analogue scale from before to after 4 weeks’ intake of boysenberry juice.

		Pre-intake	Post-intake	*P* value
Cold sensation (mm)	24 °C	37.6 ± 10.8	31.5 ± 9.7	0.994
	18 °C	88.2 ± 4.1	82.0 ± 7.2	
	Δ	50.6 ± 10.7	50.5 ± 8.7	

Data represent the mean ± SEM. Δ represented by subtracting the value at 24 °C from the value at 18 °C. The two groups were compared by a paired *t* test. The raw data are shown in Supplementary Data S4.

## Discussion

This open-label nonrandomized single-arm before-and-after study investigated the effects of intake of BoyJ, which contains BoyACs, on BAT in healthy adults over 20 years old. To our knowledge, this is the first study to examine the association between BoyACs and human skin surface temperature of BAT activity indicator. The results suggest that intake of BoyJ for 4 weeks may activate BAT in the scv area.

In humans, BAT is known to exist in scv, interscapular, perinephric, and lower axillary areas, and this study evaluated BAT activity by measuring thermogenesis in the scv region. The gold standard for measuring BAT activity is to observe glucose uptake with fluorodeoxyglucose (FDG)-positron emission tomography/computed tomography (FDG-PET/CT), but this method is invasive because it exposes participants to radiation^4,18^. Recently, a method that assesses skin surface temperature with an infrared thermography camera was reported as a simple and less invasive method for measuring BAT activity that shows similar results to FDG-PET/CT^19,20^. Therefore, to minimize the burden on study participants we chose to use an infrared thermography camera to measure BAT activity.

After 4 weeks of BoyJ consumption, skin surface temperatures, Tscv, and Tch did not change significantly, but Tscv-ch increased significantly. Subtracting the skin temperature of the non-BAT region in the upper chest from the scv BAT region has been reported to increase the sensitivity and specificity of BAT detection, so we think that significant differences were obtained only Tscv-ch^20,21^. On the other hand, skin temperature in the scv area is affected by blood flow. The decrease in hand temperature induced by cold exposure was suppressed after ingestion of BoyJ. The peripheral skin temperature is affected by skin blood flow^22^, we do not know whether this result was due to a change in skin blood flow or to BAT activation. BoyACs have also been reported to improve vascular function^23,24^. In addition, the effect of BoyACs on BAT function is assumed to be mediated through changes in vascular function^15^. Therefore, future research needs to evaluate the relationship between BAT function and vascular function. Some researchers have negative opinions about the use of skin surface temperature measurement as a method to evaluate BAT activation in individuals with a thick layer of subcutaneous fat^25^. Therefore, BAT activity should be evaluated in various ways, and future studies should consider using FDG-PET/CT.

We observed a positive correlation between Δ fat oxidation (post-intake – pre-intake) and BMI, but BoyJ intake did not significantly change EE, fat oxidation, or carbohydrate oxidation. BMI is known to be negatively correlated with BAT activity^26^. BAT produces heat predominantly through fat oxidation upon cold stimulation^27^. Blackcurrant anthocyanins only increased fat oxidation in human with a high BMI during exercise, suggesting that the effect was on adipocyte in the legs^28^. Therefore, we hypothesize that BoyP may contribute to increased fat oxidation in humans with higher BMI via BAT. In this study, the participants had a mean BMI of 20.9 ± 0.6, which is lower than the reference range defined by the Japan Society for the Study of Obesity^29^. When all participants were included, the effect size of fat oxidation was very small, suggesting that it would be difficult to show a significant difference. On the other hand, power calculations indicated that the effect size would increase by including only participants with a higher BMI. To achieve a statistical power of 0.8 with a two-sided significance level of 5% for this subgroup, the necessary sample size was estimated to be *N* = 42. These calculations indicate that the difference would be significant if sufficient participants were included. These results indicate the need to perform studies in individuals with higher BMIs.

Previous reports have pointed out the importance of carbohydrate loading before assessing BAT function^30,31^. BAT generates heat by taking up large amounts of glucose from the bloodstream and utilizing BAT free fatty acids as substrates. However, blood glucose levels are low in a fasting state, which may mean that changes in substrate utilization due to thermogenesis cannot be quantified. Our study was performed under fasting conditions, and consequently, the measurements of substrate oxidation of BAT may not have been accurate. Therefore, future research should conduct assessments under carbohydrate-loaded conditions.

The activity of BAT may be regulated by sex hormones^32^, and the positivity rate of BAT detected by FDG-PET/CT is reported to be higher in women than in men^33^. The present study included women, but it did not consider their menstrual cycle, which may have influenced the results. Therefore, future study designs should take menstrual cycle status into consideration.

BoyJ consumption had no significant effect on VAS assessments of the cold sensation. The result suggests that ingestion of BoyACs may not affect cold sensation.　The measurements scores were 88.2 ± 4.1 mm before cold exposure and 82.0 ± 7.2 mm afterwards, indicating that the cold sensation may have reached a plateau after 1 h of cold exposure. Therefore, the results are difficult to interpret, and future studies should evaluate the cold sensation over time.

## Conclusion

The study suggests that consumption of BoyAC-containing beverages for 4 weeks by healthy adults over 20 years of age increases skin surface temperature in the scv-BAT region. Although we did not observe any effect of cold exposure on systemic energy metabolism, we found a positive correlation between BMI and increased fat oxidation. Therefore, a placebo-controlled study in individuals with higher BMI is warranted to evaluate the effects of BoyACs on BAT.

## Limitations

This study has some limitations. First, because it was an open-label nonrandomized single-arm before-and-after study, the possibility of investigator bias or a placebo effect in the participants cannot be excluded. Second, the results may have been affected by sample size because only 10 individuals were included. Third, we used skin surface temperature as an indicator of BAT activity, which may not be as accurate as FDG-PET/CT measurements. And last, BAT is known to be affected by seasons, and seasonal effects cannot be excluded as the protocol^34^. This study may have been influenced by the menstrual cycle of the female participants. This study was conducted under fasting conditions, so it is possible that substrate oxidation in BAT may not have been accurately evaluated.

## Methods

### Study design

We used an open-label nonrandomized single-arm before-and-after study design. The study was approved by the Ethical Review Committee of Niigata University (approval number: 2020 − 0332) and performed in accordance with the Declaration of Helsinki and the Japanese Ministry of Health, Labor and Welfare’s Ethical Guidelines for Medical and Biological Research Involving Human Subjects. Prior to the start of the study, the study was registered in the UMIN Clinical Trials Registry (registration number: UMIN000043476).

### Participants

Participants were healthy adult males and females over 20 years old. We informed participants about the purpose, methods, expected effects, and possible adverse effects of the study and obtained their voluntary written consent to participate. Healthy men and women older than 20 years were eligible to participate if they were free from chronic diseases; gave written informed consent to participate; did not consume any drugs, Health foods that are approved as Specific Health Foods, Nutritional functional foods or Foods with Function Claim in Japan, or supplements; did not have allergies; and were not pregnant.

### Test food

The test food was boysenberry juice (BoyJ, Bourbon Corporation). Participants consumed BoyJ every day for 4 weeks at 100 ml per dose. Participants consumed 100 ml/day of the test food, i.e., boysenberry juice (BoyJ, Bourbon Corporation), for four weeks; the consumption time was not specified.

The BoyACs in the BoyJ were analyzed by high-performance liquid chromatography (HPLC)^35^. For the HPLC analysis, BoyJ was dissolved in 25 ml of 2% hydrochloric acid methanol and centrifuged (9000 rpm, 10 min). Then, 10 ml of the supernatant was scaled up to 20 ml with 10% phosphoric acid solution to obtain the sample for HPLC. The samples were injected into an HPLC instrument (Shimadzu Corporation) connected to an HPLC column (ZORBAX Extend-C18, 4.6 mm × 250 mm, 5 μm, Agilent Technologies). Eluents A (water and formic acid in a ratio of 10:90) and B (water, methanol, acetonitrile, and formic acid in a ratio of 40:22.5:22.5:10) were added, eluted with a gradient (0 min, 14% B; 20 min, 14% B; 25 min, 100% B; 30 min, 100% B), and measured at 535 nm. Cyanidin-3-glucoside (Nagara Science Corporation, code: 639-43451) was used as the standard. The four detected BoyACs (cyanidin-3-2-glucosylglucoside, cyanidin-3-2-glucosylrhamnosylglucoside, cyanidin-3-glucoside, and cyanidin-3-6- rhamnosylglucoside) were calculated as cyanidin-3-glucoside equivalents^14^. The composition of BoyJ is shown in Table 5; the juice was found to contain 61 mg of BoyACs in 100 ml.

**Table 5:** Nutritional values and boysenberry anthocyanin content of boysenberry juice.

	100 ml BoyJ
Energy content, kcal	32.0
Protein, g	0.5
Fat, g	< 0.01
Carbohydrate, g	7.2
Sodium, mg	2.0
Anthocyanins^a^	
Cyanidin-3-2-glucosylglucoside, mg	27.6
Cyanidin-3-2-glucosylrhamnosylglucoside, mg	18.5
Cyanidin-3-glucoside, mg	12.8
Cyanidin-3-6- rhamnosylglucoside, mg	2.1
Total anthocyanins (BoyACs), mg	61.0

^a^Quantitative values were calculated as cyanidin-3-glucoside equivalents.

### Assessments

**Fig. 5:**
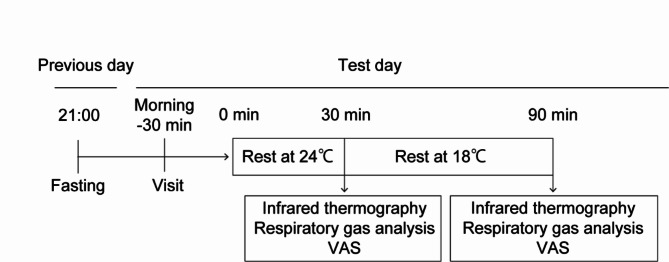
Test schedule for the study. The subjects were fasted from 21:00 on the day before the test, and infrared thermographic cameras, respiratory gas analysis, and visual analogue scale (VAS) were measured before and after cold exposure at 18 °C.

Assessments were performed at Niigata University, Niigata, Japan, in June and July 2021. Compliance was confirmed by asking participants to record their boysenberry juice intake, special activities, and any physical symptoms in a diary. Participants visited the University before and after 4 weeks of BoyJ consumption and were instructed not eat or drink anything except water after 9 p.m. on the day before the study visit. At each visit, the participants changed into a tank top with an open neck, shorts, and socks. Then, they rested for 30 min in a room set at 24 °C ± 1 °C, and their skin surface temperature was captured by an infrared thermographic camera. Subsequently, respiratory gas analysis was performed and participants rated their cold sensation on a visual analogue scale (VAS). Afterwards, participants were subjected to mild cold exposure for 1 h in a climate chamber set at 18 °C ± 1 °C. At the end of the hour, while the participants were still in the chamber, infrared thermographic imaging and respiratory gas analysis were repeated and participants completed another VAS. An overview of the test schedule is shown in Fig. 5.

#### Infrared thermographic camera

An infrared thermal camera (Testo 885, Testo K.K. Co. Ltd.) was used to measure the skin surface temperatures on the upper body and back of the hands, and the temperatures were averaged for the supraclavicular (Tscv), upper chest (Tch), and wrist to fingertip (Thand) areas. To determine the activity of BAT, the non-BAT region (Tch) was subtracted from the BAT region (Tscv) to obtain Tscv-ch. To evaluate thermogenesis by cold stimulation, ΔTscv, ΔTch, ΔTscv-ch, and ΔThand were calculated by subtracting the value before cold exposure from values after cold exposure.

#### Respiratory gas analysis

To evaluate energy metabolism, respiratory gas analysis was performed with the Douglas bag method, and energy expenditure (EE), fat oxidation, and carbohydrate oxidation were calculated with the following formulas^36,37^: energy expenditure (kcal/min) = (3.9 × VO_2_ + 1.1 × VCO_2_) × 1.44; carbohydrate oxidation (g/min) = 4.55 × VCO_2_ − 3.21 × VO_2_; and fat oxidation (g/min) = 1.67 × VO_2_ − 1.67 × VCO_2_.

Δ EE, Δ fat oxidation, and Δ carbohydrate oxidation were calculated by subtracting the value before cold exposure from values after cold exposure.

#### VAS

The VAS was used to evaluate the cold sensation. Participants were shown a straight line on which *not feeling cold* was the reference point (0 mm) and *feeling very cold* was the maximum value (100 mm) and were asked to draw a line that corresponded to their cold sensation; the length of the line was defined as the cold sensation. Δ cold sensation was calculated as the change in the length of the line from after to before cold exposure.

### Statistical analysis

The study is a preliminarily clinical trial, the required sample size was not estimated, but the number of subjects was set at 10 from similar study^38^. Data are shown as mean ± SE. A box-and-whisker plot was used to display the maximum and minimum values (range of whiskers), 25th and 75th percentiles (boxes), and median (solid line), and a dot plot was used to display the data of individual participants. A paired *t* test was used to analyze the difference in values before and after BoyJ intake. In order to evaluate correlations between data in an exploratory manner, correlations were determined by the Pearson correlation test. A post-hoc power analysis was performed. The significance level was set at 5% two-tailed. SPSS 28.01 (IBM) was used for statistical analysis.

## Electronic supplementary material

Below is the link to the electronic supplementary material.

Supplementary Material 1

## Data Availability

All data analyzed in this study are included in Supplementary Data.

## References

[1] Ricquier, D. & Bouillaud, F. Mitochondrial uncoupling proteins: From mitochondria to the regulation of energy balance. *J. Physiol.* **529 Pt 1**, 3–10. 10.1111/j.1469-7793.2000.00003.x (2000). 11080246 10.1111/j.1469-7793.2000.00003.x PMC2270181

[2] Lidell, M. E. Brown adipose tissue in human infants. *Handb. Exp. Pharmacol.* **251**, 107–123. 10.1007/164_2018_118 (2019). 29675580 10.1007/164_2018_118

[3] Ballinger, M. A. & Andrews, M. T. Nature’s fat-burning machine: brown adipose tissue in a hibernating mammal. *J. Exp. Biol.*10.1242/jeb.162586 (2018). 10.1242/jeb.162586 PMC6919643 29514878

[4] Cypess, A. M. et al. Identification and importance of Brown adipose tissue in adult humans. *N. Engl. J. Med.* **360**, 1509–1517. 10.1056/NEJMoa0810780 (2009). 19357406 10.1056/NEJMoa0810780 PMC2859951

[5] Lee, P., Swarbrick, M. M. & Ho, K. K. Brown adipose tissue in adult humans: A metabolic renaissance. *Endocr. Rev.* **34**, 413–438. 10.1210/er.2012-1081 (2013). 23550082 10.1210/er.2012-1081

[6] Liu, X., Zhang, Z., Song, Y., Xie, H. & Dong, M. An update on brown adipose tissue and obesity intervention: Function, regulation and therapeutic implications. *Front. Endocrinol. (Lausanne)*. **13**, 1065263. 10.3389/fendo.2022.1065263 (2022). 36714578 10.3389/fendo.2022.1065263 PMC9874101

[7] Hankir, M. K. & Klingenspor, M. Brown adipocyte glucose metabolism: a heated subject. *EMBO Rep.* **19**, e46404. 10.15252/embr.201846404 (2018). 30135070 10.15252/embr.201846404 PMC6123662

[8] Berbée, J. F. et al. Brown fat activation reduces hypercholesterolaemia and protects from atherosclerosis development. *Nat. Commun.* **6**, 6356. 10.1038/ncomms7356 (2015). 25754609 10.1038/ncomms7356 PMC4366535

[9] Yoneshiro, T. et al. Age-related decrease in cold-activated brown adipose tissue and accumulation of body fat in healthy humans. *Obes. (Silver Spring)*. **19**, 1755–1760. 10.1038/oby.2011.125 (2011). 10.1038/oby.2011.125 21566561

[10] Saito, M. & Okamatsu-Ogura, Y. Thermogenic brown fat in humans: Implications in energy homeostasis, obesity and metabolic disorders. *World J. Mens Health*. **41**, 489–507. 10.5534/wjmh.220224 (2023). 36792089 10.5534/wjmh.220224 PMC10307652

[11] Sun, L. et al. Capsinoids activate brown adipose tissue (BAT) with increased energy expenditure associated with subthreshold 18-fluorine fluorodeoxyglucose uptake in BAT-positive humans confirmed by positron emission tomography scan1. *Am. J. Clin. Nutr.* **107**, 62–70. 10.1093/ajcn/nqx025 (2018). 29381803 10.1093/ajcn/nqx025

[12] Kalupahana, N. S., Goonapienuwala, B. L. & Moustaid-Moussa, N. Omega-3 fatty acids and adipose tissue: Inflammation and browning. *Annu. Rev. Nutr.* **40**, 25–49. 10.1146/annurev-nutr-122319-034142 (2020). 32543947 10.1146/annurev-nutr-122319-034142

[13] Silvester, A. J., Aseer, K. R. & Yun, J. W. Dietary polyphenols and their roles in fat browning. *J. Nutr. Biochem.* **64**, 1–12. 10.1016/j.jnutbio.2018.09.028 (2019). 30414469 10.1016/j.jnutbio.2018.09.028

[14] Furuuchi, R., Yokoyama, T., Watanabe, Y. & Hirayama, M. Identification and quantification of short oligomeric proanthocyanidins and other polyphenols in boysenberry seeds and juice. *J. Agric. Food Chem.* **59**, 3738–3746. 10.1021/jf104976n (2011). 21391678 10.1021/jf104976n

[15] Furuuchi, R. et al. Endothelial SIRT-1 has a critical role in the maintenance of capillarization in brown adipose tissue. *iScience*. **25**, 105424. 10.1016/j.isci.2022.105424 (2022). 36388988 10.1016/j.isci.2022.105424 PMC9641227

[16] Sivamaruthi, B. S., Kesika, P. & Chaiyasut, C. The influence of supplementation of anthocyanins on obesity-Associated comorbidities: Aconcise review. *Foods*. 10.3390/foods9060687 (2020). 10.3390/foods9060687 PMC7353506 32466434

[17] Han, S. et al. Cyanidin-3-O-glucoside regulates the expression of Ucp1 in Brown Adipose tissue by activating Prdm16 gene. *Antioxidants*. **10**, 1986 (2021). 34943089 10.3390/antiox10121986 PMC8750179

[18] Cypess, A. M., Haft, C. R., Laughlin, M. R. & Hu, H. H. Brown fat in humans: Consensus points and experimental guidelines. *Cell. Metab.* **20**, 408–415. 10.1016/j.cmet.2014.07.025 (2014). 25185947 10.1016/j.cmet.2014.07.025 PMC4155326

[19] Law, J. et al. Thermal imaging is a noninvasive alternative to PET/CT for measurement of brown adipose tissue activity in humans. *J. Nucl. Med.* **59**, 516–522. 10.2967/jnumed.117.190546 (2018). 28912148 10.2967/jnumed.117.190546 PMC5868502

[20] Nirengi, S. et al. An optimal condition for the evaluation of human brown adipose tissue by infrared thermography. *PLoS One*. **14**, e0220574. 10.1371/journal.pone.0220574 (2019). 31449537 10.1371/journal.pone.0220574 PMC6709909

[21] Jang, C. et al. Infrared thermography in the detection of brown adipose tissue in humans. *Physiol. Rep.*10.14814/phy2.12167 (2014). 10.14814/phy2.12167 PMC4255799 25413316

[22] Cheung, S. S. Responses of the hands and feet to cold exposure. *Temp. (Austin)*. **2**, 105–120. 10.1080/23328940.2015.1008890 (2015). 10.1080/23328940.2015.1008890 PMC4843861 27227009

[23] Furuuchi, R. et al. Boysenberry polyphenol inhibits endothelial dysfunction and improves vascular health. *PLoS One*. **13**, e0202051. 10.1371/journal.pone.0202051 (2018). 30106986 10.1371/journal.pone.0202051 PMC6091942

[24] Matsusima, A. et al. Acute and chronic flow-mediated dilation and blood pressure responses to daily intake of boysenberry juice: Apreliminary study. *Int. J. Food Sci. Nutr.* **64**, 988–992. 10.3109/09637486.2013.812617 (2013). 23848379 10.3109/09637486.2013.812617

[25] Gatidis, S. et al. Is it possible to detect activated brown adipose tissue in humans using single-time-point Infrared thermography under thermoneutral conditions? Impact of BMI and subcutaneous adipose tissue thickness. *PLoS One*. **11**, e0151152. 10.1371/journal.pone.0151152 (2016). 26967519 10.1371/journal.pone.0151152 PMC4788460

[26] Vijgen, G. H. et al. Brown adipose tissue in morbidly obese subjects. *PLoS One*. **6**, e17247. 10.1371/journal.pone.0017247 (2011). 21390318 10.1371/journal.pone.0017247 PMC3044745

[27] M, U. D. et al. Postprandial oxidative metabolism of human brown fat indicates thermogenesis. *Cell. Metab.* **28**, 207–216e203 (2018). 10.1016/j.cmet.2018.05.020 10.1016/j.cmet.2018.05.020 29909972

[28] Willems, M. E. T., Banic, M., Cadden, R. & Barnett, L. Enhanced walking-induced fat oxidation by New Zealand blackcurrant extract is body composition-dependent in recreationally active adult females. *Nutrients*. 10.3390/nu14071475 (2022). 10.3390/nu14071475 PMC9002771 35406087

[29] Miyazaki, S. Himansyou shinryou guideline 2016. *Nippon Naika Gakkai Zasshi*. **107**, 262–268. 10.2169/naika.107.262 (2018).

[30] Van Schaik, L. et al. Both caffeine and *Capsicum annuum* fruit powder lower blood glucose levels and increase brown adipose tissue temperature in healthy adult males. *Front. Physiol.* **13**, 870154. 10.3389/fphys.2022.870154 (2022). 36017333 10.3389/fphys.2022.870154 PMC9395699

[31] Van Schaik, L., Kettle, C., Green, R. A., Irving, H. R. & Rathner, J. A. Using a combination of indirect calorimetry, infrared thermography, and blood glucose levels to measure brown adipose tissue thermogenesis in humans. *J. Vis. Exp.*10.3791/64451 (2023). 37335125 10.3791/64451

[32] Kaikaew, K., Grefhorst, A. & Visser, J. A. Sex differences in brown adipose tissue function: Sex hormones, glucocorticoids, and their crosstalk. *Front. Endocrinol. (Lausanne)*. **12**, 652444. 10.3389/fendo.2021.652444 (2021). 33927694 10.3389/fendo.2021.652444 PMC8078866

[33] Ouellet, V. et al. Outdoor temperature, age, sex, body mass index, and diabetic status determine the prevalence, mass, and glucose-uptake activity of 18F-FDG-detected BAT in humans. *J. Clin. Endocrinol. Metab.* **96**, 192–199. 10.1210/jc.2010-0989 (2011). 20943785 10.1210/jc.2010-0989

[34] Au-Yong, I. T., Thorn, N., Ganatra, R., Perkins, A. C. & Symonds, M. E. Brown adipose tissue and seasonal variation in humans. *Diabetes*. **58**, 2583–2587. 10.2337/db09-0833 (2009). 19696186 10.2337/db09-0833 PMC2768171

[35] Cassinese, C. et al. New liquid chromatography method with ultraviolet detection for analysis of anthocyanins and anthocyanidins in *Vaccinium myrtillus* fruit dry extracts and commercial preparations. *J. AOAC Int.* **90**, 911–919 (2007). 17760327

[36] Weir, J. B. New methods for calculating metabolic rate with special reference to protein metabolism. *J. Physiol.* **109**, 1–9. 10.1113/jphysiol.1949.sp004363 (1949). 15394301 10.1113/jphysiol.1949.sp004363 PMC1392602

[37] Frayn, K. N. Calculation of substrate oxidation rates in vivo from gaseous exchange. *J. Appl. Physiol. Respir Environ. Exerc. Physiol.* **55**, 628–634. 10.1152/jappl.1983.55.2.628 (1983). 6618956 10.1152/jappl.1983.55.2.628

[38] Yoneshiro, T. et al. Recruited brown adipose tissue as an antiobesity agent in humans. *J. Clin. Invest.* **123**, 3404–3408. 10.1172/jci67803 (2013). 23867622 10.1172/JCI67803 PMC3726164

